# Invasive and noninvasive methods for studying pulmonary function in mice

**DOI:** 10.1186/1465-9921-8-63

**Published:** 2007-09-14

**Authors:** Thomas Glaab, Christian Taube, Armin Braun, Wayne Mitzner

**Affiliations:** 1 Department of Pulmonary Medicine, III. Medical Clinic, Johannes Gutenberg-University, Mainz, Germany; 2 Fraunhofer Institute of Toxicology and Experimental Medicine (ITEM), Hannover, Germany; 3 Division of Physiology, Bloomberg School of Public Health, Johns Hopkins University, Baltimore, Maryland 21205, USA

## Abstract

The widespread use of genetically altered mouse models of experimental asthma has stimulated the development of lung function techniques in vivo to characterize the functional results of genetic manipulations. Here, we describe various classical and recent methods of measuring airway responsiveness in vivo including both invasive methodologies in anesthetized, intubated mice (repetitive/non-repetitive assessment of pulmonary resistance (R_L_) and dynamic compliance (C_dyn_); measurement of low-frequency forced oscillations (LFOT)) and noninvasive technologies in conscious animals (head-out body plethysmography; barometric whole-body plethysmography). Outlined are the technical principles, validation and applications as well as the strengths and weaknesses of each methodology. Reviewed is the current set of invasive and noninvasive methods of measuring murine pulmonary function, with particular emphasis on practical considerations that should be considered when applying them for phenotyping in the laboratory mouse.

## Background

The widespread use of genetically altered mouse models of experimental asthma has stimulated the development of lung function techniques in vivo to characterize the functional results of genetic manipulations. The ability to determine in vivo the respiratory function in laboratory mice is of great interest because of the prominent role played by these animals in biomedical, pharmacological and toxicological research. Mice are, at present, the preferred species used as an experimental model of allergic airway disease. This is largely due to a number of advantages including a well characterized genome and immune system, short breeding periods, the availability of inbred and transgenic strains, suitable genetic markers, the ability to readily induce genetic modifications and pragmatically, relatively low maintenance costs. The development of viable mouse models has largely contributed to a better understanding of the pathomechanisms underlying allergic airway inflammation and airway hyperresponsiveness (AHR) [1-3].

To fully explore the value of mouse models of experimental asthma, however, it is necessary to develop sensitive physiological methodologies that allow the quantitative assessment of airway responsiveness in intact organisms. Measurement of pulmonary function in mice clearly presents significant challenges due to the small size of their airways. In recent years, considerable progress has been made in developing valid and suitable measures of mouse lung function. Accordingly, several different invasive and noninvasive lung function techniques have been developed to characterize the phenotype of experimental models of lung disease [4-7]. Table 1 lists some of the principal advantages and limitations of invasive and noninvasive lung function methods.

**Table 1:** Principal advantages and drawbacks of invasive and noninvasive methods

Method	Pros	cons
Invasive	• sensitive and specific analysis of pulmonary mechanics	• technically demanding (instrumentation of the trachea, technical equipment)
	• based on physiological principles	• need for anesthesia and tracheal instrumentation
	• intact anatomical relationships in the lung	• time-consuming
	• bypassing of upper airway resistance, controlled ventilation, and local administration of aerosols via the tracheal tube	• no repetitive measurements in tracheostomized animals
	• ease of broncho-alveolar lavage samplings	• expertise in handling
	• repetitive and long-term measurements in orotracheally intubated mice	
	• applicable to the assessment of obstructive and restrictive* lung disorders (*requires additional hard- and software)	
noninvasive	• quick, easy-to-handle	• no direct assessment of pulmonary mechanics
	• repetitive and/or longitudinal measurements of airway responsiveness in the same animal	• prone to artifacts (movements, temperature)
	• normal breathing pattern with no need for anesthesia or tracheal instrumentation	• contribution of upper airway resistance (changes of glottal aperture, nasal passages)
		• uncertainty about the exact magnitude and localization of bronchoconstriction

It is important to recognize that each approach represents a compromise between accuracy, noninvasiveness, and convenience. As a result, a correlation exists between the invasiveness of a measurement technique and its precision [8]. The less invasive a measurement, the less likely it is to produce consistent, reproducible and meaningful data.

Invasive monitoring of lung function using parameters such as pulmonary resistance (R_L_) or dynamic compliance (C_dyn_) is the classical method for accurate and specific determination of pulmonary mechanics. R_L _is the sum of airway (Raw) and tissue (Rti) resistance, which are fairly comparable at normal breathing rate. Drawbacks of conventional invasive methodologies particularly include the surgical instrumentation of the trachea thus often excluding the practicality of repeated measurements. Modifications of the invasive approach involving orotracheal intubation, however, now have enabled repetitive monitoring of pulmonary mechanics in anesthetized, spontaneously breathing mice [9,10]. This approach still requires anesthesia as well as a good deal of technical skill to achieve reproducible consistency.

Even more detailed measurements of pulmonary mechanics can be obtained with the low-frequency forced oscillation technique (LFOT) [4,11]. In mice, LFOT is applied in anesthetized, paralyzed, tracheostomized animals to measure the complex input impedance (Z) of the lungs. The low-frequency impedance (Z) reflects the characteristically different frequency dependencies of the airway and tissue compartments. One of the major advantages of this approach is the ability to differentiate between airway and tissue mechanics in the lung.

To circumvent the significant technological challenges associated with direct measurements of pulmonary mechanics in mice, more convenient but less specific noninvasive plethysmographic methods have been studied in conscious animals [4,5,10,12,13].

This report attempts to review some of the invasive and noninvasive technologies currently used for measuring pulmonary function in intact mice with special attention to practical considerations. This review reflects our own practical experience with several different currently used lung function methods in mice. In this context, we describe the different technologies including their experimental validations, practical applications, as well as the feasibility and limitations of each methodology.

### Invasive methods for studying pulmonary function in mice

Techniques used to directly measure pulmonary mechanics in mice represent the "gold standard", but generally require anesthesia, intubation and expertise in handling.

### Determination of pulmonary resistance (R_L_) and dynamic compliance (C_dyn_) in tracheostomized and mechanically ventilated mice

The classical approach to determine lung function in mice is the measurement of pulmonary resistance (R_L_) and dynamic compliance (C_dyn_) in response to non-specific bronchoconstrictors. In 1988 Martin et al. demonstrated the feasibility of R_L _and C_dyn _measurement in anesthetized, tracheotomized and mechanically ventilated mice [14]. To assess R_L _and C_dyn _determination of transpulmonary pressure and flow are required. In mice the chest wall has been shown to present little mechanical load compared to the mechanical load of the lung [15], unless there is some pathology of the chest wall. Thus direct measurement of transpulmonary pressure is generally not mandatory [16]. Tidal flow is commonly derived from the differentiation of the volume signal. R_L _and C_dyn _can then be calculated by fitting an equation of motion to the measurements of pressure, flow and volume [4]. In this equation, P_TP _= V × R_L_+ VT/C_dyn_, P_TP _is transpulmonary pressure (or in the mouse ≈ transrespiratory pressure), V is tidal airflow, R_L _is pulmonary resistance, V_T _is tidal volume, and C_dyn _is the dynamic pulmonary compliance. The invasive measurement of R_L _and C_dyn _by body plethysmography normally requires surgical instrumentation of the trachea in anesthetized animals. It is common to use pentobarbital sodium (70–90 mg/kg) administered intraperitoneally as anesthetic because it normally provides an adequate depth of anesthesia for at least 30 minutes. Alternative anesthetic regimens in mice have been described [6,7]. It is important not to disturb and agitate the animal beforehand, as this may impact the quality of the subsequent measurement. Useful reflexes to ensure that an adequate depth of anesthesia has been attained include loss of the righting reflex (lost during the onset of anesthesia) and of the toe-pinch reflex (lost during medium to deep anesthesia). If the animal attempts to withdraw its limb, then it is not sufficiently anesthetized and should be administered an additional dose (~10–20% of the initial dose).

Determination of R_L _and C_dyn _not only provides the classical determination of airway responsiveness, but also provides a more detailed insight into pulmonary mechanics. R_L _reflects both narrowing of the conducting airways and parenchymal viscosity. In contrast C_dyn _is considered to primarily reflect the elasticity of the lung parenchyma, but is also influenced by surface tension, smooth muscle contraction and peripheral airway inhomogeneity. Numerous methods for determining R_L _and C_dyn _have been described in anesthetized and instrumented mice [4,5,7]. One option is to use a (mass-constant) body plethysmography box with the tracheal cannula leading out of the plethysmograph [17,18]. When mechanical ventilation is indicated, tracheostomy is usually performed for endotracheal intubation of the deeply anesthetized animal. The surgically exposed trachea is viewed directly and the incision is made in the upper third of the trachea to allow proper insertion of the cannula and to avoid measuring artifacts.

The tracheostomy tube can then be attached to a four-way connector, where two ports of the connector are attached to the inspiratory and expiratory sides of a ventilator and the remaining tube to a pressure transducer that measures tracheal pressure. Ventilation should then be set at a rate comparable to normal breathing (around 150 breaths/min, tidal volume ≈ 8–10 ml/kg) with a positive end-expiratory pressure (PEEP) of 2–5 cm H_2_O. It is important to use PEEP in mice even with the chest closed, since functional residual capacity (FRC) in conscious mice is normally maintained with active inspiratory muscle tone that is minimal or eliminated in the anesthetized animal [16]. Lung volume changes must be assessed by calibrating the plethysmographic pressure. To stabilize the volume signal for thermal drift the body plethysmograph chamber can be connected to a large bottle filled with copper gauze.

To assess airway responsiveness, cholinergic bronchoconstrictive agents such as methacholine (MCh) are administered to the animal at increasing doses either by aerosol inhalation or systemically by intravenous administration via the tail or jugular vein. Airway responsiveness is assessed either as the change in R_L _compared to baseline or as the peak response after challenge. Before each series of challenge doses the lung should be briefly hyperinflated to standardize the volume history. Measurements are made of the absolute values of the responses of C_dyn _and R_L _and as a percentage of baseline, determined from an initial vehicle challenge.

The key advantage of the invasive approach is the reproducible and precise assessment of transient changes in pulmonary mechanics in mice. The insertion of a tracheal tube also avoids measurement of changes in the upper airways, and provides the opportunity for taking broncho-alveolar lavage (BAL) samples after lung function measurements. Disadvantages of conventional invasive measurements include surgical tracheostomy thus precluding repeated measurements, the need for anesthesia, mechanical ventilation and expertise in handling.

### Repetitive assessment of R_L _and C_dyn _in orotracheally intubated mice

As outlined above, the utility of invasive determination of murine lung function is generally limited by several factors. Recent methodological advances, however, have improved the ability to measure lung mechanics on repeated occasions [19]. These modifications involving direct laryngoscopy have now enabled repetitive determination of pulmonary mechanics (R_L _and C_dyn_) in combination with local aerosol administration via an orotracheal tube in intact animals [9,10,20].

**Figure 1:**
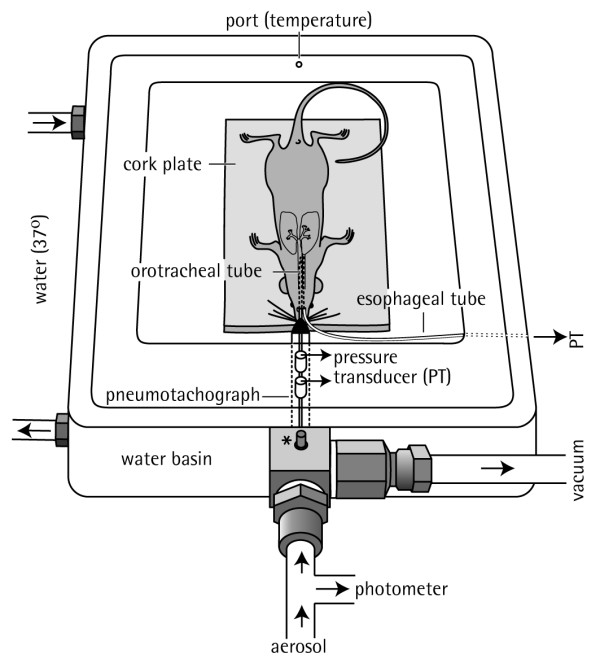
**Diagram of the plethysmograph used for pulmonary function testing of anesthetized, orotracheally intubated mice**. A thermostat-controlled water basin (37°C) built in the plethysmograph chamber ensured a body temperature of 34–35°C as measured by rectal thermometer. Defined aerosol concentrations of methacholine, as measured by an aerosol photometer, were delivered into the airways via the orotracheal tube. For calculation of pulmonary resistance (R_L_), transpulmonary pressure (P_TP_) was recorded via an esophageal tube, and tidal flow was determined by a pneumotachograph attached directly to the orotracheal tube. PT, pressure transducer. Taken from [10] with permission.

With one of these approaches, intubation is done with a standard 20G × 32 mm (1 1/4 inch) teflon cannula (e.g. Abbocath^®^-T cannula, Abott, Ireland) in anesthetized mice that are suspended by their upper incisors from a rubber band and the midthorax held by an elastic band on a 65° incline Plexiglas support to facilitate intubation. We have made positive experience using anesthesia plus analgesia with 20–30 mg/kg etomidate and 0.05 mg/kg fentanyl given intraperitoneally (i.p) with minimal supplementations as required or volatile anesthesia with halothane 1.5 % plus propofol 70 mg/kg i.p. Paralysis is not mandatory. A metal laryngoscope (length 12 cm plus an additional 1.8 cm at an angle of 135°, with 0.3 cm) is used as a tool to allow visualization of the tracheal opening which is transilluminated below the vocal cords by a halogen light source. The direct visualization of the trachea allows gentle insertion of the cannula into the tracheal opening [19,21]. Orotracheal intubation of the anesthetized mouse takes about five minutes and has also been successfully applied in mouse cardiac surgery [21]. Alternatively, a Seldinger technique has been described using a 0.5 mm optical light fiber as an introducer over which the cannula is slid down into the proximal trachea [22]. The intubated, spontaneously breathing animal is then placed in supine position in a thermostat-controlled whole-body plethysmograph (Figure 1). The orotracheal tube is directly attached to a pneumotachograph/differential pressure transducer unit to record tidal flow. To measure transpulmonary pressure (PTP), a water-filled polyethylene (PE)-90 tubing is inserted into the esophagus to the level of the midthorax and attached to a pressure transducer. R_L _and C_dyn _are calculated over a complete respiratory cycle with an integration method over flows, volumes and pressure [10,23]. The resistance of the orotracheal tube (0.63 cm H_2_O·s·ml^-1^) is subtracted from R_L _recordings.

This approach was validated in several groups of BALB/c mice [10]. The results showed that dose-related increases in R_L _and C_dyn _to inhaled cholinergic challenge with MCh were reproducible over short and extended intervals without causing significant cytological alterations in the BAL fluid or relevant histological changes in the proximal trachea and larynx regardless of the number of orotracheal intubations.

A key advantage of this method which combines orotracheal intubation via direct laryngoscopy and local administration of aerosols directly into the lung is the repetitive assessment of classical measures of pulmonary mechanics to defined inhalation challenges in intact individual mice. Because the orotracheal cannula is tapered, a tight seal develops as it is inserted into the proximal trachea. This enables use of this method in spontaneously breathing as well as in mechanically ventilated mice. Orotracheal intubation further offers the opportunity to collect BAL samples in vivo on multiple occasions in the same animals [24]. Limitations include the need for anesthesia, instrumentation of the trachea and expertise in handling.

### Low-frequency forced oscillation technique

Another approach for invasive assessment of airway function in mice is the low-frequency forced oscillation technique (LFOT). The LFOT was derived from similar techniques used in humans and larger animals and produces estimates of lung impedance (Z) which can be considered the most detailed measurement of pulmonary mechanics currently available [4,8,11,25,26].

Different parts of the impedance frequency spectrum reflect different parts of the respiratory system. Impedance data can be further analyzed using the Constant Phase Model which provides a suitable assessment of pulmonary mechanics [27]. Fitting the Constant Phase Model to oscillatory data allows airway and tissue mechanical components to be distinguished.

Until recently, little was known about lung impedance of mice, particularly because of technical difficulties of measuring lung impedance precisely. Lung impedance consists of two parts. One part of impedance, resistance (R), describes essentially the resistance of the conducting airways (Raw) and tissue (Rti). The second part of impedance, referred to as reactance (X), reflects respiratory compliance (1/elastance) and characterizes the lung parenchyma. The contribution of the inertance (I) of the gas in the murine airways, however, is only significant at frequencies ≥ 20 Hz. The main advantage of this approach for measuring lung function, compared to the classical methods of assessing airway resistance and dynamic compliance, is that the more sophisticated mathematical models may better represent the complexity of the intact lung.

Two different methods have been developed to assess lung impedance in small animals. One technique uses a small plastic wave tube that is placed into the trachea and is attached to a loudspeaker [4,28].

The properly miniaturized wave tube has a precisely known geometry and material constant. During the measurement ventilation is paused and the setting is switched from the ventilation to the measurement circuit. The loudspeaker produces an oscillatory flow through the tube and lung impedance is assessed from flow and pressure measurements along the tube. From the pressure spectra along the tube lung impedance can be assessed [29]. This technique is particularly useful for the precise measurement in very young mice, where other techniques such as the piston pump oscillator may be critical [30]. The second method uses a computer-controlled piston pump. This system not only allows for mechanical ventilation of the animal but also for precise frequency and amplitude control of the applied oscillations. The constant phase model is then fit to the data obtained from the multiple frequencies simultaneously applied at the airway opening, thereby enabling determination of the airway and lung tissue impedance. This model involves three independent variables: airway resistance (R) as a marker of central airway resistance, tissue damping (G) is related to tissue resistance and reflects the dissipative properties, while tissue elastance (H) describes the elastic properties of the lung tissue.

LFOT correlates well with classical measures of lung resistance and has been successfully used to assess airway responsiveness in mouse models of allergic airway disease [4,15,25,28,31-33]. The computer-controlled ventilator also allows the assessment of quasi-static compliance. As with other invasive techniques, the animals need to be anesthetized, tracheally intubated and then connected to the computer-controlled ventilator (e.g. set at a rate of 150 breaths per minute and a tidal volume of 10 ml/kg), with application of 2–5 cm H_2_O PEEP. Mice can then be challenged with bronchoconstrictors by inhalation or via intravenous routes. It should be considered that while LFOT can be employed during apnea only, paralysis is not mandatory in anesthetized mice.

The main advantage of this technique is the detailed analysis of airway function and particularly the clear distinctions between central airways and more peripheral changes. This approach, however, also shares similar disadvantages with other invasive techniques as shown in Table 1. In addition, at least for assessing airway hyperresponsiveness it is still unclear what additional value lung impedance recordings provide over simpler measures of pulmonary mechanics [34].

### Noninvasive methods for studying pulmonary function in mice

Noninvasive plethysmographic methods of monitoring pulmonary function are preferred for long-term serial study designs as well as for screening large numbers of conscious mice. In many instances, a combination of invasive and noninvasive techniques is required to fully understand the physiologic significance of a respiratory phenotype.

### Barometric whole-body plethysmography

**Figure 2:**
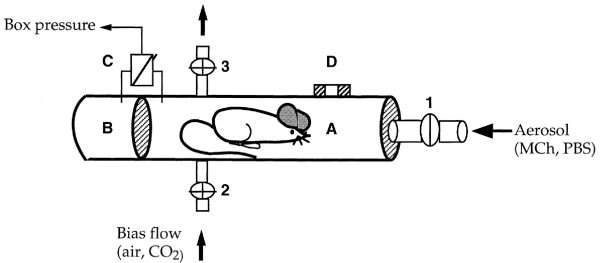
**Diagram of the barometric whole-body plethysmograph **(taken from [35] with permission). (A) Main chamber containing the animal (B) connected to a pressure transducer (C) which is also connected to the reference chamber (B). (D) Pneumotachograph. Main inlet for aerosol. The bias airflow at 0.2 L/min was discontinued during aerosol challenges.

In barometric whole-body plethysmography mice are placed in a closed chamber and the pressure fluctuations that occur during the breathing cycle are recorded [35]. In contrast to invasive measurements of airway function animals are neither anesthetized nor instrumented and are relatively unrestrained. The major benefit of this noninvasive technique is that repetitive measurements can be done in the same mouse. Using a pressure transducer the pressure differences between the main chamber of the plethysmograph where the animal is placed and a reference chamber are assessed (Figure 2).

From this pressure time curve several parameters can be determined including breathing frequency, inspiratory and expiratory time as well as the maximum box pressure during inspiration and expiration. None of these variables is specific nor sensitive enough for being a suitable marker of airway responsiveness. From the box pressure signal during inspiration and expiration, and the timing comparison of early and late expiration, a dimensionless parameter called "enhanced pause" (Penh) has been calculated. It is notable that we do not refer to the as yet non-validated method of measuring Penh in freely moving mice.

To monitor responsiveness mice are exposed to a nebulized bronchoconstrictor such as MCh and changes in Penh are recorded for ~2–5 minutes for each aerosol challenge. Usually the response is expressed as fold increase of Penh for each MCh concentration compared with Penh values after an initial buffer challenge with the aerosolized vehicle.

Early studies in mice and other species showed a correlation between changes in Penh following methacholine challenge and lung function parameters determined by invasive lung function measurements and the technique has been widely used [18,36-39]. Based on this early work and because of the convenient handling of the animals, this method gained popularity in many research labs. An increasing amount of observations, however, have now cast doubt on the validity of Penh to reflect airway narrowing. Several reports found discrepancies in the amount of airway responsiveness when comparing Penh to conventional parameters of pulmonary mechanics [40-42]. Further evaluation of Penh demonstrated that events completely unrelated to lung mechanics such as humidification and warming of inspired gas, hyperoxia, and the timing of ventilation, have a major effect on the measurement [31,41]. These more careful and theoretical findings have thus led to a justifiable scepticism for using Penh as a reliable marker of airway obstruction [43-45].

Nevertheless, in principle and consistent with current cautionary warnings, Penh may be useful for gross screening of overall lung function in small animals [43]. Seen by itself, however, Penh says nothing about airway responsiveness and researchers who use it should corroborate the measurements with parallel, independent direct measurements of pulmonary mechanics [5,7,44,45]. Pros and cons of this method are summarized in Table 2.

**Table 2:** Pros and cons of noninvasive barometric whole-body plethysmography

pros	cons
• minimal restraint of the animal	• enhanced pause as an empirically derived value with unclear physiological relevance
	• influenced by a number of factors unrelated to bronchoconstriction
	• potential to overestimate or underestimate the real degree of airway responsiveness
	• data need to be confirmed by invasive methodology

### Head-out body plethysmography

Recent emphasis on the benefits of noninvasive technology has renewed interest in analyzing expiratory tidal flow patterns as a tool in the assessment of airway obstruction. Although noninvasive measurement of murine respiratory function has virtually become synonymous with the widely used barometric whole-body plethysmography method [35], some other noninvasive methods have been described [13,46-48].

**Figure 3:**
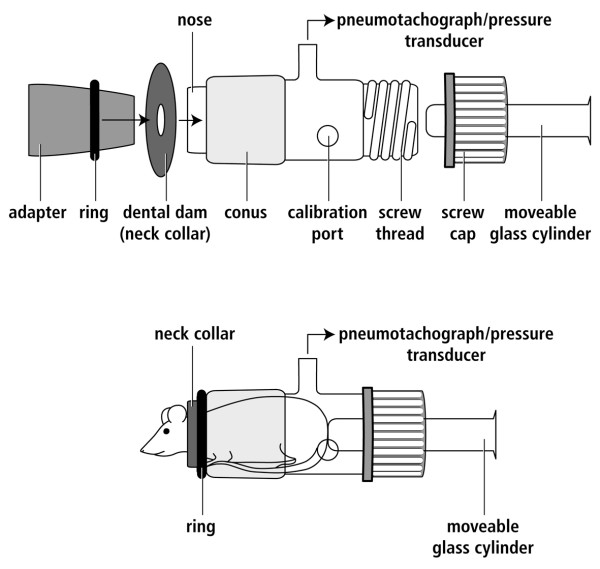
**Schematic drawing of the head-out body plethysmograph**. The figure illustrates the attachment of the neck collar (made of dental dam with a central hole of 7–8 mm for a 20–25 g mouse) to the plethysmograph. The adapter is put in the front opening of the plethysmograph and a viscoelastic ring is slipped over the fixed rubber dam at the nose of the plethysmograph thus fixing the collar. The conscious animal is then placed in the glass plethysmograph and attached via the conus to a ventilated head exposure chamber. A moveable glass cylinder built in the screw cap enables atraumatic positioning of the mouse. Volume calibration (1–1.5 ml air) of the plethysmograph (front and back opening sealed) is done before each measurement. Before data collection, mice are allowed to acclimatize for at least about 10 minutes in the body plethysmographs.

**Figure 4:**
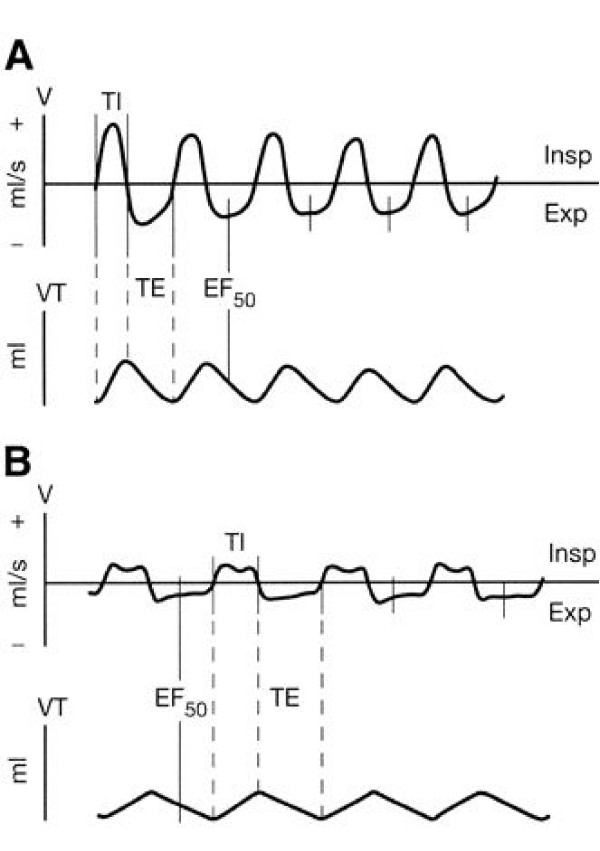
**Characteristic modifications to the normal breathing pattern in conscious BALB/c mice**. *A*: normal breathing pattern of BALB/c mice breathing room air. *B*: characteristic pattern of airway obstruction during aerosol challenge with MCh, illustrating the decline in EF_50_. *A *and *B*, *top *tracings: pneumotachograph airflow signals. *A *and *B*, *bottom *tracings: corresponding integrated VT signal. A horizontal line at zeroflow separates inspiratory (Insp; upward; +) from expiratory (Exp; downward; -) airflow. V, tidal flow. VT, tidal volume. TI, time of inspiration. TE, time of expiration. Figure taken from [49] with permission.

The noninvasive measurement of midexpiratory flow (EF_50_) as measured by head-out body plethysmography (Figure 3) was first described as an appropriate instrument to measure airway responsiveness in conscious mice by Alarie et al [48]. With this method, airway constriction induces characteristic changes in the tidal flow pattern, which are best revealed by a decrease in tidal midexpiratory flow (EF_50_, [ml/s]) (Figure 4). The change in EF_50 _is typically linked with a reduction in tidal volume (VT), breathing rate (f) and prolonged expiratory time (TE). EF_50 _can be determined with a glass head-out body plethysmography system. Animals are gently placed in the body plethysmographs while the head of each animal protrudes through a neck collar into a ventilated head exposure chamber. Aerosols can be delivered directly through the head exposure chamber. Tidal flow measurement is made with a calibrated pneumotachograph and a differential pressure transducer attached to the top port of each body chamber. The amplified and digitized flow signals are integrated with time to obtain tidal volume. From these signals several standard respiratory parameters, including tidal volume, breathing frequency, time of inspiration and expiration, and EF_50 _can be derived from software analysis.

Validation studies in mice have demonstrated that the decline in EF_50 _to inhaled cholinergic and allergic challenge closely reflects the decreases in simultaneously recorded pulmonary conductance (G_L _= 1/R_L_) and dynamic compliance (C_dyn_) [10]. The EF_50 _method has been applied in several experimental situations, including animal models of experimental asthma, post-pneumonectomy, hyperoxia, and to study the effects of airborne toxicologic agents [31,39,48-54]. Advantages of this approach are its noninvasiveness and its allowing simple, rapid and repeatable measurements of several conscious animals at a time. Moreover, EF_50 _is based on physiological principles and has a physical meaning [ml/s] that is directly related to airway resistance, thus enabling quantitative interpretation of airway changes between animals [55]. In principle, head-out body plethysmography as described by Alarie et al. also enables evaluation of the sensory irritation potential of inhaled agents by recording the prolongation of the postinspiratory pause in mice [48,51].

Concerns include the uncertainty about the potential contribution of upper airway resistance. To minimize effects of restraint stress on responses, monitoring of respiratory function should not be started until animals and individual measurements have settled down to a stable level. Because it has been shown that EF_50 _may underestimate the magnitude of bronchoconstriction [9,10] it is still unclear how much this limits its use in detecting less pronounced changes in airway hyperresponsiveness. Accordingly, when such circumstances are present, EF_50 _measurements should be confirmed by more direct assessments of pulmonary resistance. Table 3 summarizes the pros and cons of EF_50 _measurements.

**Table 3:** Pros and cons of noninvasive tidal midexpiratory flow measurement

pros	cons
• based on physiological principles	• underestimation of the magnitude of airway responsiveness as compared with direct measures of pulmonary mechanics
• acceptable agreement with simultaneous invasive measurements of pulmonary mechanics	• restraint by neck collar
• physical meaning enables comparability of data from animal to animal	

## Conclusion

In this manuscript we have tried to provide a review of the advantages and disadvantages of different methods of assessing pulmonary function in mice. Although mice may be far from perfect models of human lung disease, the advantages of using mouse models has made them the choice for many experimental studies, e.g. experimental asthma. In these models measuring lung function and particularly airway responsiveness is a major outcome parameter. To this end it is critically important to have suitable methods of phenotyping lung function. Although many of the methodologies for measuring pulmonary function have been developed, there are important limitations and considerations such as expertise, technical difficulty of the procedure, and costs, which should be recognized when applying them in the mouse. Unfortunately, at the present time, there is no gold standard for measuring lung function in mice, since none of the available methods is optimal in all regards. Some investigations require more detailed measurement of the individual mechanical properties, and these studies normally require invasive determination of pulmonary mechanics. The ability to make longitudinal measurements in intact conscious mice, however, allows investigators to make use of more powerful statistics with smaller numbers of animals. We have discussed the merits of several of these approaches that may be useful for investigators requiring this approach. In particular in situations where the measurements are applied to develop a potential therapeutic or clinical trial design, these should always be confirmed by the more conservative invasive methodologies.

## Abbreviations (Table 4)

**Table 4:** 

**Parameter**	**Abbr**.	**Description**
lung resistance	R_L_	quantitatively assesses the level of obstruction in the lungs and comprises the resistance of the conducting airways (R_aw_) and tissue (R_ti_)
lung conductance	G_L_	reciprocal of lung resistance (1/R_L_)
dynamic compliance	C_dyn_	primarily reflects the elasticity of the lung parenchyma, but is also affected by surface tension, smooth muscle constriction, and peripheral airway inhomogeneities. In contrast, static compliance is measured at true equilibrium, when resistances and compliances are not uniform throughout the lung, e.g. in the absence of any motion.
methacholine	MCh	non-specific cholinergic bronchoconstrictor used to assess airway responsiveness
elastance	E	captures the elastic rigidity of the lungs.
reactance	X	reflects respiratory compliance (1/elastance) and characterizes the lung parenchyma
input impedance	Z	expresses the combined effects of resistance, compliance and inertance as a function of frequency.
inertance	I	represents the inertive properties of the gases in the airways. The majority of I resides in the central airways bypassed by the tracheal cannula. Inertance can be ignored in the mouse below 20 Hz.
tissue damping	G	is closely related to tissue resistance and reflects the dissipative properties of the lung tissues.
tissue elastance	H	reflects the elastic properties of the lung tissues.
enhanced pause	Penh	is a unitless, empirical measurement derived from box pressure signals during inspiration and expiration and the timing comparison of early and late expiration and is used as a non-invasive measure of bronchoconstriction.
tidal midexpiratory flow	EF_50_	is defined as the tidal flow at the midpoint of expiratory tidal volume and is used as a non-invasive measure of airway constriction.
positive end-expiratory pressure	PEEP	is the amount of pressure above atmospheric pressure present in the airway at the end of the expiratory cycle. PEEP improves gas exchange by preventing alveolar collapse, recruiting more lung units, and increasing functional residual capacity.

## Competing interests

The author(s) declare that they have no competing interests.

## Authors' contributions

TG and CT conceived of the review and drafted the manuscript, AB helped to draft the manuscript, WM helped to draft, discuss and revise the manuscript. All authors read and approved the final manuscript.
